# Molecular imaging of bacterial biofilms—a systematic review

**DOI:** 10.1080/1040841X.2023.2223704

**Published:** 2023-07-15

**Authors:** S. W. G. van Hoogstraten, C. Kuik, J. J. C. Arts, B. Cillero-Pastor

**Affiliations:** a Laboratory for Experimental Orthopaedics, Department of Orthopaedic Surgery, CAPHRI, Maastricht University Medical Centre, Maastricht, the Netherlands; b Maastricht MultiModal Molecular Imaging Institute (M4I), Maastricht University, Maastricht, the Netherlands; c Department of Biomedical Engineering, Orthopaedic Biomechanics, Eindhoven University of Technology, Eindhoven, the Netherlands; d Department of Cell Biology-Inspired Tissue Engineering, The MERLN Institute for Technology-Inspired Regenerative Medicine, University of Maastricht, Maastricht, the Netherlands

**Keywords:** Molecular imaging, bacterial biofilm, mass spectrometry, spectroscopy, multimodal imaging

## Abstract

The formation of bacterial biofilms in the human body and on medical devices is a serious human health concern. Infections related to bacterial biofilms are often chronic and difficult to treat. Detailed information on biofilm formation and composition over time is essential for a fundamental understanding of the underlying mechanisms of biofilm formation and its response to anti-biofilm therapy. However, information on the chemical composition, structural components of biofilms, and molecular interactions regarding metabolism- and communication pathways within the biofilm, such as uptake of administered drugs or inter-bacteria communication, remains elusive. Imaging these molecules and their distribution in the biofilm increases insight into biofilm development, growth, and response to environmental factors or drugs. This systematic review provides an overview of molecular imaging techniques used for bacterial biofilm imaging. The techniques included mass spectrometry-based techniques, fluorescence-labelling techniques, spectroscopic techniques, nuclear magnetic resonance spectroscopy (NMR), micro-computed tomography (µCT), and several multimodal approaches. Many molecules were imaged, such as proteins, lipids, metabolites, and quorum-sensing (QS) molecules, which are crucial in intercellular communication pathways. Advantages and disadvantages of each technique, including multimodal approaches, to study molecular processes in bacterial biofilms are discussed, and recommendations on which technique best suits specific research aims are provided.

## Introduction

**Figure 1.**
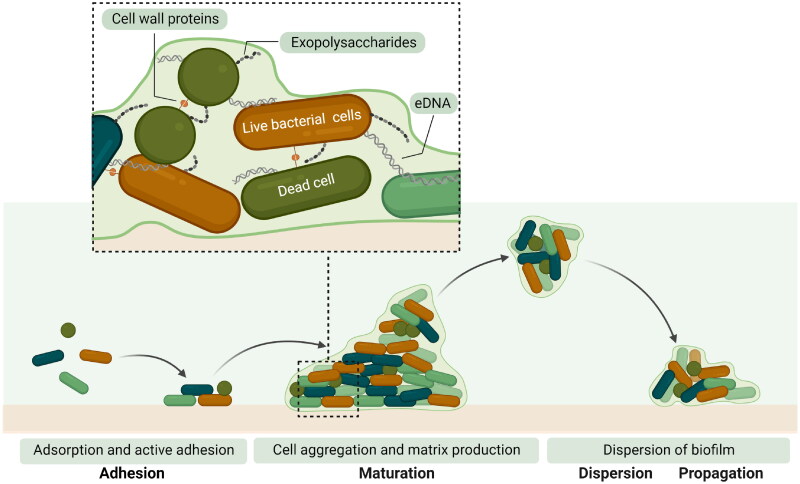
The stages of biofilm formation. Bacteria adhere to the biomaterial by chemical and physical forces followed by biofilm maturation where the EPS is produced and bacterial cells aggregate. For *Staphylococcus aureus and Staphylococcus epidermidis*, biofilm formation includes the production of polysaccharides, intercellular adhesion, and the release of extracellular DNA. A mature biofilm can disperse and propagate to a new infection location (Created with biorender.com).

Bacterial biofilms are complex surface-attached communities of bacteria in a self-produced matrix mainly consisting of extracellular polymeric substances (EPS), including proteins, polysaccharides, extracellular DNA, and other molecules (Bjarnsholt 2013; Khatoon et al. 2018; Muhammad et al. 2020). Bacterial biofilms are microbial communities that colonize and grow on surfaces. Biofilms are a natural state of bacteria, but when colonized on medical implants, such as sutures, catheters, and joint replacement implants, they cause severe infections. The formation of a biofilm is a complex-multistage process typically classified into three stages (Figure 1) (Arciola et al. 2018). During the initial state, planktonic bacteria adhere to a surface *via* physical (van der Waals) and chemical forces. The maturation stage involves the production of signalling molecules by the bacterial cells, expression of biofilm-specific genes, and intercellular communication. Bacteria in a biofilm can sense the distance and size of neighbouring bacterial clusters using quorum-sensing (QS), which guides them to produce clusters that can efficiently interact with adjacent cells (Gu et al. 2013). The microcolonies increase in size and thickness, and the bacterial cells produce EPS, which encloses the microcolony community and stabilizes the biofilm network (Khatoon et al. 2018). In the dispersion stage, the biofilm matrix can disrupt, and bacteria can migrate to other body sites *via* haematogenous spread.

Over 80% of all microbial infections in humans include biofilm formation, and the resistance of biofilms to antimicrobial agents is at the root of many life-threatening infections (Lewandowski and Beyenal 2014). In healthcare, 60–80% of all infections are associated with some type of implanted device or biomaterial, including orthopaedic implants, prosthetic heart valves, and urinary catheters, where the pathogen causing the infection usually originates from the patient’s skin or the surgical site (Bryers 2008). Among many other biofilm-forming pathogens, *Staphylococcus aureus* and *Staphylococcus epidermidis* are the leading cause of hospital-acquired infections (Donlan 2000).

In the clinic, bacterial infections involving biofilms are often chronic and difficult to treat, as the matrix-embedded bacterial cells in a biofilm can tolerate antibiotics and host defense systems (Bjarnsholt et al. 2008; Bjarnsholt, Jensen, et al. 2009; Bjarnsholt, Tolker-Nielsen, et al. 2009). Bacteria existing within a biofilm are protected by the EPS, so bacterial resistance to antibiotics is increased up to 10 000-fold compared to planktonic bacteria (Luo et al. 2021). This strong resistance results from the biofilm matrix acting as a diffusion barrier, delaying penetration of antimicrobial agents into the biofilm, as well as the decreased growth and multiplication rate of the biofilm-embedded bacteria, limiting drug uptake (Arts and Geurts 2017). In addition, debridement or removal of the biofilm from the human body without excessive side damage is difficult due to the strong mechanical integrity of the biofilm matrix (Gordon et al. 2017). Furthermore, the range of effective antibiotic drugs for biofilm treatment is extremely limited, which forms a significant problem together with the increasing threat of antimicrobial resistance in bacteria (Nadeem et al. 2020). Besides biofilms in the clinical field, bacterial biofilms impose a major problem worldwide in industry and environmental settings, for example, in the food industry, and in wastewater processing. The adverse effects of biofilms are responsible for a global economic burden of $4 trillion (USD), where the main part of these costs are related to the medical and human health sector (Cámara et al. 2022).

Therefore, strategies to prevent biofilm formation and treat mature biofilms have been a major topic in infection research over the past decades, as these strategies are essential to combat the high hospitalization and mortality rates of biofilms (Chen et al. 2013; Mishra et al. 2020). Many studies have been dedicated to developing new treatment options for mature biofilms, targeting cell-cell communication pathways or altering EPS composition and structure, leading to an increased penetration depth of administered drugs. Anti-adhesion or bactericidal coatings have been developed to reduce bacterial attachment on implants to prevent biofilm formation (Desrousseaux et al. 2013; Srinivasan et al. 2021). With the rising occurrence of antimicrobial resistance, biofilm research has shifted focus to eliminate the use of antibiotics to prevent and treat biofilms. However, clinical and biomedical biofilms remain a major problem, and treating infection caused by biofilm-forming bacteria remains extremely challenging. An in-depth analysis of the biofilm is required to gain insight into new areas of biofilm treatments and elucidate the failure mode of existing biofilm treatments.

Tracking the effect of an environmental change on biofilm compounds or the ability to visualize alterations in the structural hierarchy of biofilm molecules can lead to new angles in biofilm treatment. Visualization of metabolic pathways can be used to assess drug uptake and efficiency. Spatiotemporal mapping of active inter-cellular communication signalling molecules can be used to optimize the administration method and timing of anti-biofilm drugs or identify novel combination-based therapies. Furthermore, a detailed visualization of cell-material interactions on a molecular level will elucidate the possibilities and pitfalls of new antimicrobial material technologies. Currently, in biofilm research, the most frequently used biofilm visualization methods include general morphological and topographical techniques, such as scanning electron microscopy (SEM) and atomic force microscopy (AFM). Staining, such as crystal violet and safranin-O that quantify bacterial cells or biomass and show their distribution, are frequently used. Confocal laser scanning microscopy (CLSM) combined with live/dead staining, is also often employed in biofilm research and shows a three-dimensional biofilm shape and distribution of dead and living bacterial cells. Besides these techniques, molecular imaging techniques, defined as techniques able to spatially map specific molecules and molecular classes in a bacterial biofilm, are not as commonly used due to several challenges: the high hydration level, complex structure, and the ability of biofilms to grow on many different host materials in various environments. However, molecular imaging techniques offer great potential in biofilm research.

Molecular imaging techniques, including MSI, Raman spectroscopy, and CLSM, have been optimized to spatiotemporally track the distribution and transport of molecules and ions in live biofilms, providing a better understanding of the composition and distribution of metabolites regulating micro-processes of the biofilm over time. An in-depth analysis of biofilm composition, structure, formation, cell-cell communication, metabolism, drug delivery, and response to environmental stress can significantly contribute to preventing and treating clinical biofilms (Hua et al. 2015). Therefore, this systematic review provides an overview of the literature that used molecular imaging modalities for biofilm visualization and characterization, with a critical perspective on the included literature. The strengths and applications of different modalities in biofilm research will be highlighted, and the most critical methodological developments will be described. Furthermore, this systematic review will elaborate on how the included molecular imaging techniques can deepen our understanding of bacterial biofilm formation, maturation processes, and treatment effects and where the potential of (combined) molecular image acquisition in biofilm research lies.

## Methods

The Preferred Reporting Items for Systematic Reviews and Meta-Analysis (PRISMA) methodology was followed for the literature search and selection. The online databases PubMed and EMBASE were used to search the literature. The search was conducted on August 26, 2022. In PubMed, the following search string was used: ((“Molecular Imaging”[Mesh]) OR (molecular imaging)) AND (((“Biofilms”[Mesh]) OR (biofilm)) OR (biofilms)). The search strategy used for the EMBASE search is shown in Table S1. Individually and blinded, two researchers (SH, CK) evaluated all articles using “Rayyan QCRI.” Articles that included molecular imaging of bacterial biofilms were included in further screening. Article exclusion criteria used were: reviews, abstracts only, text not in English, no molecular imaging, and no imaging of biofilm. Also, articles focussed on marine or ecological biofilms without any clinical correlation or a pathogen not clinically relevant were excluded under the label “not medical related.” A third researcher (BCP) evaluated the conflicting articles.

## PRISMA results and data extraction

**Figure 2.**
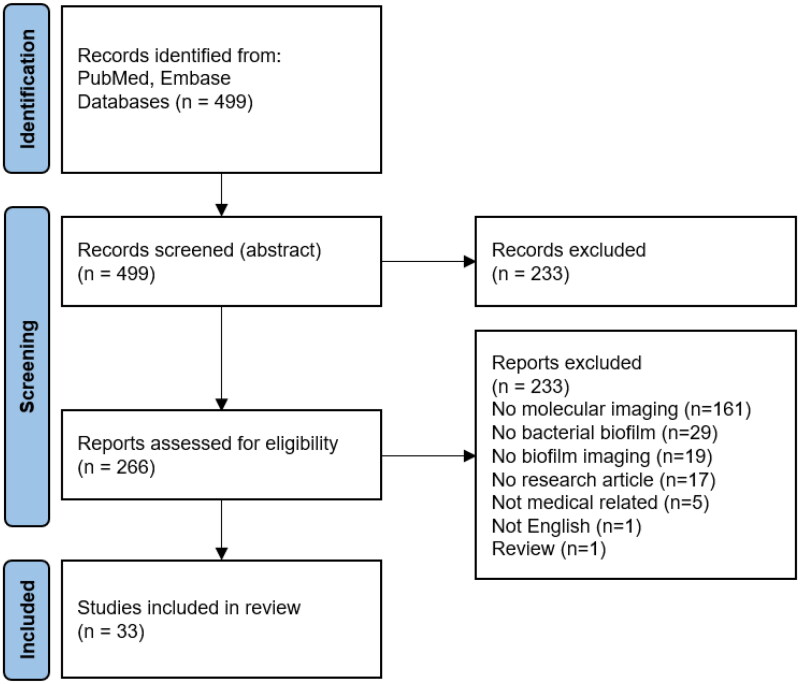
PRISMA flow diagram, screened abstracts, and articles. After the database search, 499 articles were identified. Based on abstract screening, 266 papers were read and assessed for eligibility. This systematic review used a total of 33 articles for qualitative analysis.

From the EMBASE and PubMed search, 499 articles were identified and screened on their abstract by the exclusion and inclusion criteria mentioned above. During the abstract screening, 233 articles were excluded. The 266 included articles were assessed for full article screening. After the full-text screening, 33 articles were considered eligible for this study (Figure 2). An overview of the most essential extracted data is shown in Table 1. In addition, relevant information was extracted from the included articles, including authors, year of publication, imaging technique employed, molecular classes or pathways that were imaged, and biofilm parameters that were imaged (Table 2). The following sections will summarize and discuss all data extracted from the included literature per imaging modality. The advantages and disadvantages of all discussed techniques are provided in an overview in Box 1. In Table 3, an overview is provided on recommended biofilm research applications for each imaging modality.

**Table 1.** Data extraction and study characteristics of selected articles: molecular classes analyzed in bacterial biofilms, sorted by an imaging technique.

Author, year	Imaging modality	Molecular class imaged
MSI [15]		
Davies et al. (2017)	SIMS	Quorum-sensing molecules
Dunham et al. (2018)	SIMS	Quorum-sensing molecules
Ding et al. (2016)	SIMS (liquid)	Quorum-sensing molecules, fatty acids, lipids, carbohydrates
Hua et al. (2014)	SIMS (liquid)	Fatty acids
Hua et al. (2015)	SIMS (liquid)	Fatty acids
Zhou et al. (2016)	SIMS (liquid)	Molecular fragments (non-specified)
Blaze et al. (2012)	MALDI	Proteins, peptides
Brockmann et al. (2019)	MALDI-2	Quorum-sensing molecules, metabolites
Brockmann et al. (2021)	IR-MALDI-2	Quorum-sensing molecules, lipids
Lukowski et al. (2021)	MetA-LDI	Proteins, peptides, lipids
Kurczy et al. (2015)	NIMS	Molecular fragments (non-specified)
Louie et al. (2013)	NIMS	Metabolites
Cui et al. (2013)	Fs-LDPI-MS	Metabolites
Zhang et al. (2020)	Cryo-OrbiSIMS	Lipids, quorum-sensing molecules, nucleobases, metabolites
Watrous et al. (2013)	nanoDESI	Proteins, peptides, lipids, fatty acids
Spectroscopy [7]		
Bodelón et al. (2016)	SERS	Metabolites
Polisetti et al. (2017)	SERS	Metabolites
Do et al. (2019)	SERS	Metabolites
Baig et al. (2016)	SERS	Metabolites, quorum-sensing molecules
Ivleva et al. (2010)	SERS	Metabolites, proteins, carbohydrates
Garg et al. (2022)	SERS	Proteins, carbohydrates, DNA
Holman et al. (2009)	SR-FTIR	Proteins, carbohydrates, DNA
Fluorescence imaging [4]		
Berk et al. (2012)	CLSM	Proteins, carbohydrates
Decker et al. (2015)	CLSM	Proteins
Narasimhan et al. (2017)	CLSM	Carbohydrates
El-Kirat-Chatel et al. (2014)	Fluorescence microscopy	Proteins
NMR [1]		
Simkins et al. (2018)	NMR	Oxygen
µCT [1]		
Keren-Paz et al. (2018)	µCT	Calcium carbonate
Multimodal [5]		
Lanni et al. (2014)	SIMS, MALDI	Metabolites, quorum-sensing molecules
Lanni et al. (2014)	SIMS, CRM	Quorum-sensing molecules, proteins, carbohydrates
Baig et al. (2015)	SIMS, CRM	Quorum-sensing molecules
Dunham et al. (2016)	SIMS, MetA-SIMS, MetA-LDI	Quorum-sensing molecules
Si et al. (2016)	MALDI, fluorescence microscopy	Metabolites

**Table 2.** Imaging specifics, biofilm characteristics, and molecular classes can be imaged per imaging modality.

Method	Dimension		Lateral resolution	Results		Molecular class						Biofilm state		References
	2D	3D		Quantitative	Qualitative	Proteins	Peptides	Lipids	Metabolites/signalling molecules	Elements	*In situ* imaging of native biofilm?			
**MSI**													
SIMS	X	X	0.1–5 µm		X		X	X	X	X	Yes (liquid SIMS only)		Seeley and Caprioli 2012; Brison et al. 2013; Sjovall et al. 2015
MALDI	X		5–50 μm^2^	X	X	X	X	X	X		No		Rzagalinski and Volmer 2017; Vaysse et al. 2017
NIMS	X		5–50 μm^2^	X	X	X	X	X	X		No		O'Brien et al. 2013
Nano DESI	X		10–100 μm^2^	X	X		X	X			Yes		Laskin and Lanekoff 2016
**Fluorescence imaging**													
CLSM	X	X	0.2 μm^2^	X	X	X	X	X	X		Yes		Baak 2002; Bohmer and Enderlein 2003
**Spectroscopy**													
SERS	X		0.2 μm	X	X	X		X	X		Yes		Ivleva et al. 2010
SR-FTIR	X		5–10 μm		X	X			X		No		Holman et al. 2009
**µCT**	X	X	0.87 μm^3^	X	X					X			Keren-Paz et al. 2018
**NMR**	X		0.2 μm^2^	X	X					X	No		Simkins et al. 2018

## Molecular imaging techniques for biofilm imaging

### Mass spectrometry imaging

MSI is a technique that allows direct spatial visualization of molecular species on various sample surfaces and enables mapping different molecular classes, such as drugs, metabolites, lipids, peptides, and proteins. MSI allows label-free molecular imaging, but labels can track targeted analytes that are difficult to ionize (Caprioli et al. 1997; Chughtai and Heeren 2010; Claes et al. 2023). With MSI, an ionization source moves along the tissue sample where molecules at specific coordinates are ionized and directed into the mass spectrometer. Mass spectra of ionized molecules are collected and combined into an image, providing spatial information of every mass-to-charge (m/z) value corresponding to a particular molecule in the tissue sample (Buchberger et al. 2018). Different ionization probes are available for MSI, the most commonly used being ion beams for secondary ion mass spectrometry (SIMS) or lasers for matrix-assisted laser desorption/ionization (MALDI). Another MS modality reported in the included articles of this review is nanospray desorption electrospray ionization (nanoDESI) MS. The type of ionization and its corresponding ionizing efficiency leads to different types of molecular classes that can be identified. In total, fifteen studies included in this review used MSI techniques for molecular imaging of a bacterial biofilm. MSI techniques are of high value in biofilm research, as they provide an untargeted investigation of the molecular distribution in bacterial biofilms. Untargeted analysis can provide new information on biofilm development, cell-cell interaction, and biofilm response to modified surfaces.

#### Secondary ion mass spectrometry

SIMS is a powerful tool that provides elemental, isotopic, and molecular information with high sensitivity and high spatial resolution, down to sub-nm in-depth and ∼50 nm lateral resolution (Zhou et al. 2016). A primary advantage of SIMS, when compared to MALDI or DESI, is the high spatial resolution allowing single-colony level analysis and the ability to create a reconstruction of three-dimensional images of the sample using sectioning or sputtering depth profiling techniques. Davies et al. created such 3D images and were able to simultaneously image endogenous and exogenous biofilm compounds, giving biochemical information on biofilm composition (Davies et al. 2017). Antibiotics and biofilm metabolites were imaged simultaneously, which is an essential tool in future research to gain an understanding of how biofilms respond to an antibiotic challenge. Furthermore, the study by Davies et al. (2017) showed another important feature of SIMS, as biofilms were grown and imaged on both conductive glass slides and in *ex vivo* pig lung tissue, offering the freedom of substrates on which a biofilm can be imaged. The freedom of substrate material is limited by its conductivity, however, non-conductive substrates can also be analyzed after the application of a conductive coating. Therefore, the freedom of substrate material makes SIMS a highly valued technique in clinical biofilm research, as it can be used for *in vitro* biofilm models and more complex samples. This is particularly interesting for research on cell-material interactions, for example, in developing a new anti-biofilm surface or for a better understanding of drug efficacy in several types of tissues.

In the past, SIMS imaging has been primarily used as a qualitative technique as it is challenging to map the absolute quantity of a compound. However, Dunham et al. reported a method for quantitative imaging of small molecules in agar-based biofilms using SIMS (Dunham et al. 2018). A biofilm was grown on thin agar, dried under a stream of nitrogen, and using quadratic calibration, the surface density of each analyte was presented on a pixel-by-pixel basis, enabling quantitative comparison within and between samples. The technique is more time-consuming than traditional SIMS when analyzing a broad range of analytes but effective for quantitative imaging of the surface density in many different sample types. Dunham et al. quantitatively imaged quinolone distribution in a 2D surface density image. Understanding and controlling this chemical communication system could lead to a broad range of medical and industrial applications (Dunham et al. 2018).

Traditionally, SIMS was used for solid samples due to the high vacuum principle of the technique. For example, all vacuum-based characterization techniques need dehydration steps using cryogenic freezing. However, water removal causes drastic changes to biofilm matrix integrity and morphology, as shown by previous studies (Hua et al. 2014). Hua et al. (2014) conducted a significant difference in detecting characteristic fatty acid fragments in hydrated versus dehydrated biofilms. Therefore, a new technique was presented to follow the hydrated state dynamics of biofilm attachment, growth, and dissociation dynamics in real-time and space, with high-resolution chemical mapping. A vacuum-compatible microfluidic reactor was used, where a biofilm grows in the reactor on a silicon nitride membrane at the liquid interface, and is imaged *in situ* with SIMS. The portability and vacuum compatibility of this method offer a valuable linkage with proteomic mass spectrometry *via* microfluidics and a non-destructive *in situ* analysis of live biofilms.

Hua et al. present 3D chemical images of hydrated biofilms and an *in situ* time and space-resolved identification of characteristic biofilm fatty acid fragments, highlighting the potential of liquid SIMS (Hua et al. 2015). Fatty acids play a crucial role in biofilm formation and dispersion and provide the support and viscosity needed to form a biofilm matrix. Logically, liquid SIMS imaging led to the first molecular detection of water clusters within the biofilm, as reported by Ding et al. (2016). Furthermore, they found that water cluster distribution changes to external environmental changes, which can affect the hydrophobicity of the biofilm (Ding et al. 2016). Moreover, they used liquid SIMS to investigate how biofilm components respond to an external environmental stressor, stressing the potential of this technique in biofilm research.

**Figure 3.**
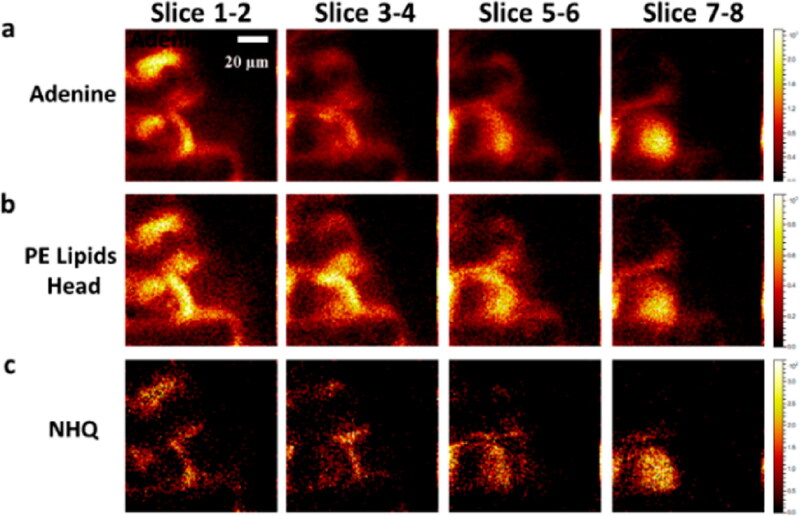
Orbitrap MS images of a frozen-hydrated *Pseudomonas aeruginosa* biofilm. (a) Adenine, a nucleic acid marker that can originate from both the bacterial cytoplasm and the extracellular DNA present in the extracellular matrix. (b) PE lipid head groups, markers for the bacterial membrane and only associated with bacterial cells and macrovesicles. (c) NHQ (5-Nitro-8-Hydroxy Quinoline) is an extracellular signalling molecule, but because of its physical properties, a high proportion is associated with the cell envelope and any macrovesicles that had been shed into the biofilm matrix. Image from Zhang et al. (2020). Permission for using the image was granted.

Liquid SIMS enables new opportunities to study biofilms in their native state, and this *in situ* molecular imaging will aid in understanding how the spatial heterogeneity and structural difference affect the microbial community activities in an unperturbed hydrated state. Continuous imaging of complex liquid samples helps to understand complex environmental processes as biofilms interact with surfaces across multiple domains (molecular to mesoscale). This real-time and space chemical molecular spatial mapping can better address the scientific and medical challenges of bacterial biofilms regarding biofilm prevention and eradication. A downside of *in-situ* liquid SIMS analysis is signal intensity. Especially the signal intensity of positive ions is too low to detect with a system for analysis at the liquid vacuum interface (SALVI). This may result from the constant release of water vapour in the aperture area and the interaction between the liquid surface and the beam current. However, Zhou et al. optimized the ion beam to increase the intensity of both positive and negative molecular ion signals, but further optimization is still needed (Zhou et al. 2016). To increase the molecular coverage and field of view, which is more than 2 μm diameter with liquid SIMS when imaging a hydrated biofilm, Zhang et al. (2020) focussed on developing the Cryo-OrbiSIMS method. They reported a method for molecular imaging of biological materials preserved in a native state using an OrbiSIMS instrument equipped with cryogenic sample handling and a high-pressure freezing protocol compatible with mass spectrometry (Zhang et al. 2020). A hybrid instrument with MS for high-speed 3D imaging and a high-field Orbitrap MS for high mass resolving power was used to visualize frozen-hydrated biofilms. They annotate 87 compounds, including nucleobases, amino acids, PE lipids, quinolones, and lactones, doubling the number of detectable biofilm molecules (Figure 3).

#### (Matrix-assisted) laser desorption ionization mass spectrometry imaging

Laser desorption ionization (LDI) mass spectrometry is a technique that uses a laser to ionize molecules on a surface. The laser will impact molecules from the surface, thereby desorbing and ionizing them. In some cases, this ionization mechanism is insufficient, and enhancing techniques, such as MALDI, can be used. Today, MALDI is a commonly applied MSI technique for tissue analysis. With traditional MALDI, a matrix is applied to the sample. The MALDI matrix consists of small molecules with an acidic functional group for proton transfer, linear conjugated π systems, and/or aromatic rings for photon absorption in the UV region. The analyte co-crystalizes with the matrix, and when impacted by a laser, the matrix molecules absorb the light and transfer the energy to the analytes.

Several types of matrices enable the ionization of certain groups of molecules, so the choice of the matrix can partially tailor the chemical specificity (Seeley and Caprioli 2012). An advantage of MALDI, when compared to SIMS, is the soft ionization nature, resulting in minimal molecular fragmentation which enables imaging of large intact biological molecules. MALDI-MSI is used for spatial imaging of biofilms and visualization of molecular processes within the biofilm by analyzing peptides, proteins, lipids, and metabolites (Blaze et al. 2012; Brockmann et al. 2019). Proteins and lipids are two primary components of the extracellular matrix encasing mature biofilms, contributing to the antimicrobial resistance of biofilms (Alim et al. 2018). Furthermore, lipids are important determinants of cell attachment to surfaces and biofilm formation (Rowlett et al. 2017). An insight into the impact of lipids on biofilm formation will increase our understanding of bacterial pathogenesis and contribute to the therapeutic field. In-depth analysis of lipids, proteins, and peptides elucidates the bacterial microbiome and bacterial ecology by investigating molecular processes (Flemming et al. 2007). When these are identified, new material technology might be developed to block or counter biomolecules involved in biofilm formation.

Blaze et al. showed the importance of MALDI-MSI for analyzing biofilms as they imaged specific proteins on the membrane produced by *Enterococcus faecalis*, a major cause of urinary tract infections with increasing antibiotic resistance (Blaze et al. 2012). Even a relatively low spatial resolution provided useful information on biofilm molecules. Specific proteins and peptides in the biofilm play a role in cell adhesion and virulence properties. The spatial localization of these molecules and the discovery of unknown proteins within intact biofilms may improve the understanding of bacterial virulence mechanisms. Brockmann et al. employed MALDI to analyze the interaction of competing microbial colonies by looking at QS molecules and rhamnolipids (Brockmann et al. 2019). An improved understanding of the metabolic exchange between two microorganisms could help in the development of potential therapies targeting specific metabolites, interfering with biofilm formation or maturation processes. By looking at the rhamnolipid distribution, centred at the interaction zone between competing bacteria, Brockmann et al. found rhamnolipids are used as a defense and attack mechanism of the bacteria (Brockmann et al. 2019).

Even though essential information about biofilm processes can be gained, MALDI has some downsides in biofilm research. When analyzing a biofilm by MALDI, only the highly abundant species present on the bacterial surfaces can be detected and identified, limiting the number of species that can be detected. Another drawback is spot-to-spot variability observed within a single analysis arising from ionization differences rather than analyte heterogeneity. This might be due to non-homogenous matrix application, detector saturation, sample charging, or ion suppression due to local differences in molecular dynamic range, only leading to the detection of a fraction of all molecules in a biofilm. Also, when imaging biofilms with MALDI, the substrate material is limited as biofilm samples are commonly grown on agar and transferred to a MALDI stainless steel target plate or conductive indium tin oxide (ITO) coated sides. The sample substrate is essential, as conductive surfaces are often required to prevent charge buildup.

In response to these drawbacks, studies have been dedicated to optimizing the MALDI methodology. Brockmann et al. enhanced the ionization efficiency of MALDI and improved the spatial resolution by using laser post-ionization (MALDI-2), which includes a second laser to ionize neutral molecules (Brockmann et al. 2019). Using MALDI-2, a higher number of small metabolites and lipids were analyzed. Also, they tested different sample preparation protocols, including steam inactivation, as safe sample handling is a frequent problem for MSI analysis. A sample preparation protocol, including steam inactivation, was tested, and bacteria were inactivated within 5 s without any other effects on biofilm structure and can be safely analyzed outside a fume hood (Brockmann et al. 2019).

Even though matrix application enables the analysis of a wide range of molecules, a major drawback of MALDI is related to the matrix. Commonly applied matrices in MALDI-MSI analysis have molecular masses like drugs and metabolites, resulting in interference and ionization competition of the matrix molecules with the analytes. The overlap of small molecules and matrix molecules in the mass spectra can be overcome by eliminating the matrix. Furthermore, higher spatial resolutions can be achieved since the size of the matrix crystals limits the spatial resolution. Also, the matrix and the matrix solvents kill bacterial cells, restricting the analysis of molecules in the native state. To benefit from the advantages of soft laser ionization without producing high-intensity matrix ions, the development of matrix-free soft LDI platforms is essential. Brockmann et al. evaluated the potential of infra-red (IR) MALDI (Brockmann et al. 2021). This method enables water to be used as a laser absorbent, eliminating the need for MALDI matrices. The IR-MALDI-2 spectra showed a lower level of chemical background, and additional metabolites, which MALDI-2 did not previously record, were detected by IR-MALDI-2.

Besides IR-MALDI-2, other laser desorption ionization modes were employed to enhance the identification of biomolecules in the low molecular weight range. For example, femtosecond laser desorption post-ionization (fs-LDPI) MSI was used by Cui et al. to visualize the spatial distribution of a biofilm, and numerous *m/z* values corresponding to metabolites were imaged (Cui et al. 2013, 2015). With fs-LDPI-MSI, ultrashort pulse lasers are used for MS imaging. Sub-100 fs laser pulses create non-resonant desorption, eliminating the need for matrix application. Also, fs laser ablation can image the sample with minimal damage, enabling same-spot analysis for depth profiling, which is impossible with traditional MALDI. However, the presented method showed implications, leaving SIMS as the MSI method of choice for the highest spatial resolution and depth profiling.

**Figure 4.**
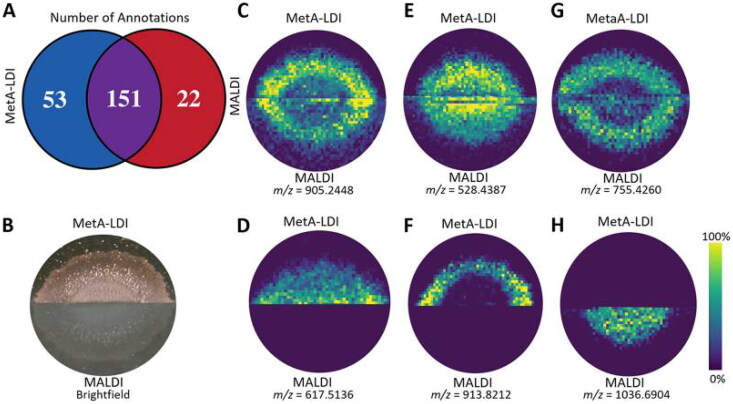
Comparison between MALDI MSI and MetA-MSI. (A) A Venn diagram illustrates a 67% overlap of annotations between both techniques. (B) A brightfield image of the colonies on agar, analyzed by both techniques. Ion images of (C) corynebactin, (D) diacylglycerol, (E) ceramide, (F) triaglycerol, (G) phosohoglyserol, and (H) surfactin C. Image from Lukowski et al. (2021). Permission for using the image was granted.

Metal-assisted laser desorption/ionization (MetA-LDI) was employed by Lukowski et al. to increase the molecular coverage for biofilm analysis (Lukowski et al. 2021). With MetA-LDI, a metal is sputter-coated onto the sample surface to help the ionization of endogenous biofilm molecules. No MALDI matrix is used, resulting in no interference in the low-mass range. Furthermore, the metal coating forms a more homogenous layer than the MALDI matrices, resulting in less analyte delocalization. The study of Lukowski et al. (2021) showed a 67% overlap of detected molecules when comparing MetA-LDI and MALDI-MSI, but each ionization technique lead to the identification of a unique subset of molecules (Figure 4). MetA-LDI identified more neutral lipids and small molecules, whereas MALDI detected more peptides. A major advantage of this method is that adding a metal coating helps ionization from electrically nonconductive substrates. This allows the investigation of biofilms grown in a broader range of *in vitro* models. Another technique developed to enhance small molecule profiling without using a MALDI matrix is nanostructure-Initiator Mass Spectrometry Imaging (NIMS). Besides better detection of small molecules, this technique has high sensitivity and low background compared to traditional MALDI-MSI due to matrix elimination. The study by Louie et al. enabled NIMS imaging of microbes grown on agar surfaces by adapting the sample transfer method to an extraction gel, and signalling molecules within a biofilm were successfully spatially mapped. Furthermore, the study by Kurczy et al. used fluorinated nanoparticles to facilitate NIMS for biofilm imaging (Kurczy et al. 2015). However, the detected mass spectra were not further identified.

#### Nanospray desorption electrospray ionization mass spectrometry

**Figure 5.**
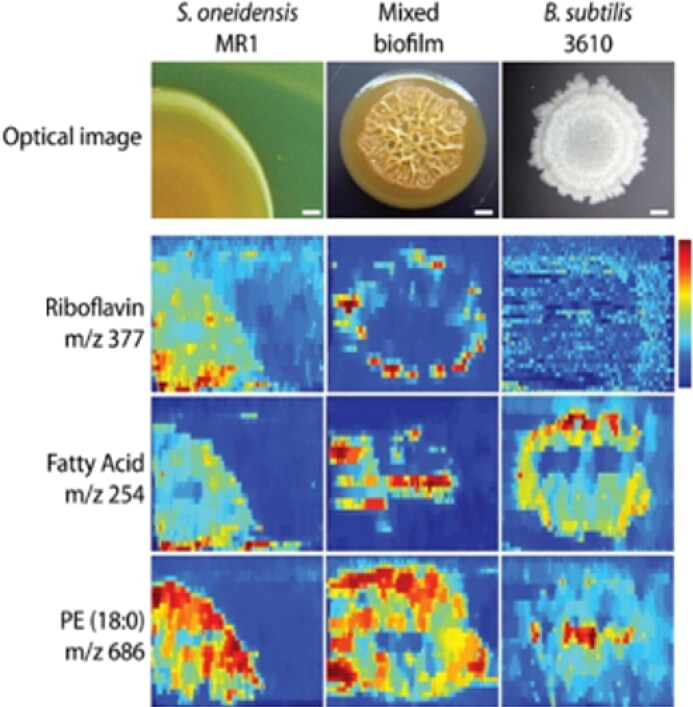
nanoDESI IMS biofilm images showing riboflavin (vitamin B12, which plays an essential role in extracellular electron transfer by *Shewanella oneidensis* MR-1), fatty acids, and phosphatidylethanolamines. Image adjusted by permission from Watrous et al. (2013).

One paper in this systematic review reported nanoDESI as a biofilm imaging modality (Watrous et al. 2013). NanoDESI is a variation on the ionization method DESI, an ambient ionization technique using an electrospray for ionization and desorption of molecules on a sample surface. In nanoDESI, two capillaries form a liquid bridge for more localized liquid extraction, enabling higher spatial resolution than conventional DESI (Li et al. 2022). DESI and nanoDESI have the unique capability of metabolic profiling of living bacterial colonies and biofilms directly from the petri dish at ambient pressure, with no sample preparation needed (Figure 5). Analysis in ambient pressure allows direct profiling without deformation or flacking of the sample caused by freezing or vacuum. Biofilms can be directly imaged from the agar plate, which is a major advantage of nanoDESI.

### Raman spectroscopy

Raman spectroscopy is an analytical technique that can be applied non-destructively and noninvasively for detecting and imaging a wide range of molecules. Compared to MSI, Raman benefits from fast acquisitions, its non-destructive nature, and minimal sample preparation (Eberhardt et al. 2015). Raman spectroscopy combines spectroscopic and optical methods based on the effect of inelastic light scattering by molecules (Ivleva et al. 2010; Desmond et al. 2018). The inelastic scattering results in molecular information on the vibrational, rotational, and other low-frequency modes of chemical bonds present in the sample, leading to informative vibrational spectra of the analyzed samples with a spatial resolution in micrometres. Raman spectra contain information on chemical compositions and biomolecular structures, including bonding situations, symmetry, and physical parameters (e.g. the length of any chemical bonds). Frequency peaks in the Raman spectra correspond to specific molecules, such as proteins, nucleic acids, carbohydrates, and lipids (Eberhardt et al. 2015). A whole-organism fingerprint can be obtained using Raman spectroscopy; therefore, the technique has a broad clinical and diagnostic application in bacterial research (Ashton et al. 2011). It can identify pathogens in complex clinical samples, and fast antibiotic resistance profiling can be performed by monitoring the effect of antibiotics on the pathogen (Pavlicek et al. 2017).

**Figure 6.**
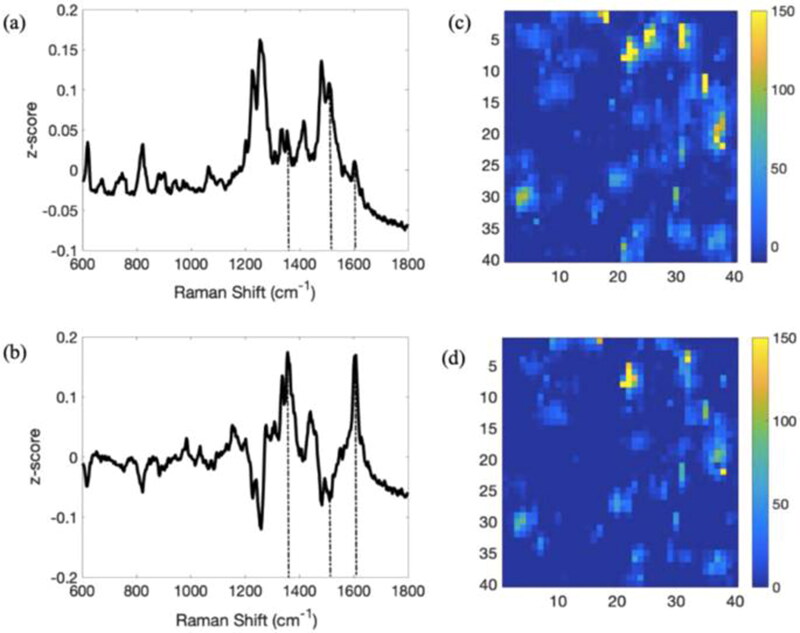
Raman spectra and SERS images of *a Pseudomonas aeruginosa* biofilm showing PYO, a QS molecule of the opportunistic human pathogenic bacterium *P. aeruginosa*. Direct detection of PYO in biofilms is crucial because PYO can provide important information about infection-related virulence mechanisms in *P. aeruginosa*. Figure from Do et al. (2019) (Do et al. 2019). Permission for using the image was granted.

Raman spectroscopy can be applied directly *in situ* in an aqueous environment and simultaneously visualize a biofilm’s chemical composition and molecular structure in its native state. However, Raman spectroscopy has only been applied in a few studies for molecular imaging of a bacterial biofilm. A downside of Raman spectroscopy compared to other molecular imaging modalities, such as fluorescence labelling, is the limited sensitivity and the fact that complex biological samples, such as biofilms, tend to be weak Raman scatterers, making it exceedingly difficult to obtain good Raman spectra without extremely long collection times (Jarvis and Goodacre 2004). There are several enhancement methods to increase the sensitivity of the Raman spectra, with the most popular being Surface-enhanced Raman scattering (SERS). SERS offers highly specific spectra for identifying multicomponent samples in a non-destructive and rapid manner. With SERS, molecules are in proximity or bound to nano-sized noble metallic compounds, which enhance Raman sensitivity significantly by localized surface plasmon resonance and charge transfer. Polisetti et al. showed increased sensitivity when using SERS with silver particles compared to conventional Raman spectroscopy (Polisetti et al. 2017). It must be kept in mind that the nanoparticles can be toxic to bacterial cells and might affect the original biofilm components. SERS is mainly used to visualize QS signalling molecules when analyzing bacterial biofilms. QS regulates gene expression in response to the accumulation of signalling molecules for cell-cell interactions. A certain “quorum” or population of bacteria excretes these molecules (Shrout et al. 2006). The Pseudomonas quinolone signals (PQS) and pyocyanin (PYO) are two essential molecules in this QS network for *Pseudomonas aeruginosa* biofilms. Direct detection of PYO in *P. aeruginosa* biofilms is crucial because PYO can provide important information about infection-related virulence mechanisms. PQS is involved in biofilm development, surface motility, and membrane vesicle formation, while PYO is an antibiotic and virulence factor in host infection (Bevers et al. 2022). Baig et al. investigated PQS and PYO molecules to study *P. aeruginosa* biofilm formation and growth processes (Baig et al. 2016). Multiple other studies reported the spatial detection of PQS and PYO by SERS (Figure 6) (Bodelón et al. 2016; Polisetti et al. 2017; Do et al. 2019). Before imaging these molecules, the Raman spectrum of the specific molecule must be obtained as a reference using the pure analyte. Therefore, Raman-based technologies are limited by targeted analysis for the identification of the imaged molecules. However, Ivleva et al. studied bacterial biofilms in an untargeted manner (Ivleva et al. 2010). In this study, bands in acquired SERS spectra were tentatively identified as different molecular groups potentially correlating to proteins, DNA, RNA, carotenoids, and lipids. To perform a SERS measurement, a bacterial culture grown in a growth medium was transferred onto silicon tile or glass slides to cultivate a biofilm, followed by the application of colloidal silver particles for enhanced sensitivity. Grag et al. demonstrated the potential of microporous multi-resonant plasmonic meshes (MMPMs) as bio-interface surface-enhanced Raman spectroscopy sensors to enable molecular profiling of bacterial biofilms (Garg et al. 2022).

### Synchrotron-radiation-based Fourier transform infra-red spectromicroscopy

Another modality within the spectroscopy field is synchrotron-radiation-based Fourier transform infra-red spectromicroscopy (SR-FTIR). FTIR spectroscopy uses polychromatic radiation to measure the excitation of molecular bonds whose relative absorbance provides an index of the abundance of various functional groups based on the usage of IR light. Absorption of IR light occurs when photon transfer to the molecule excites it to a higher energy state, resulting in molecular bond vibrations (Jamin et al. 1998; Miller and Dumas 2006). The IR spectra contain peaks representing the absorption of IR light by specific molecular bonds at specific frequencies. SR-FTIR spectromicroscopy has been used as a label-free approach to track biogeochemical changes with high sensitivity and micrometer spatial resolution in real-time. Furthermore, the infra-red beam used with SR-FTIR does not exceed the toxic limit for bacteria, so it will not alter the biofilm’s chemical or morphological nature. Therefore, SR-FTIR has been found well suited for monitoring chemical changes in bacteria during their stress-adaptive response. However, the abundance of water in biofilms has hindered SR- FTIR’s sensitivity in investigating bacterial activity and biofilms (Loutherback et al. 2015). This is in contrast to Raman spectroscopy, which is characterized by a low water background, which is beneficial for *in situ* analysis of biofilms. Furthermore, the interference of the water signal in biofilms will complicate the identification of molecules within a sample due to signal overlapping. In 2009, Holman et al. presented a method based on an open-channel microfluidic system that can circumvent the water-absorption barrier for chemical imaging of the developmental dynamics of bacterial biofilms with a spatial resolution of several micrometres (Holman et al. 2009). Holman has shown that by combining an open-channel system with SR-FTIR spectromicroscopy, a living bacteria community can be maintained on biofilm over a long period while making continuous spectroscopic measurements and chemical imaging. An open-channel microfluidic approach was used to minimize water absorption and the interference fringe problem while maintaining the functionality of microbial cells and capturing molecular information about microbial processes within biofilms over time.

### Fluorescence microscopy

Fluorescence microscopy, such as CLSM, is a traditional technique to study biofilm and EPS. CLSM permits the examination of the biofilm structure concerning the matrix composition and spatial localization of important biofilm compounds. CLSM is a powerful technique for morphological studies and clinical assessments, but it can also be used for molecular characterization of the biofilm. The principle of CLSM relies on a laser source and a scanning device based on fluorescence microscopy imaging and applies a conjugate focussing device based on traditional optical microscopy to achieve layer-by-layer scanning and sample imaging (Zhang et al. 2019). Fluorescent probe labels are used to visualize specific components in the biofilm. When used correctly, a lateral resolution as low as 200 nm can be reached depending on the wavelength of illumination used, the aperture of the objective, and the diffraction limit of light, which dictates both the maximum lateral and axial resolution (Trinh and Fraser 2015). The axial resolution is achieved by a confocal pinhole that rejects the emitted fluorescence from above and below the focal plane. This eliminates all out-of-focus light to prevent blurring of the image (Pawley 2006). Therefore, the speed, resolution, and laser power must be balanced for optimal biofilm imaging. As CLSM provides high sensitivity and non-destructive analysis, biofilms can be quantitatively analyzed in a three-dimensional manner, and the distribution of extracellular proteins, lipids, nucleic acids, polysaccharides, and many more molecules can be obtained (Möhle et al. 2007).

**Figure 7.**
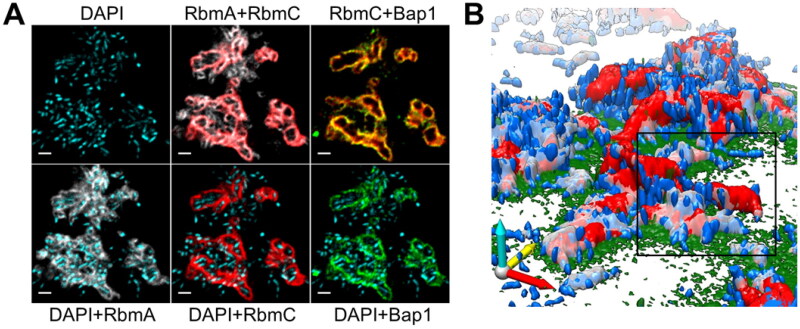
(A) CLSM images of *Vibrio cholerea biofilm* visualizing pseudo-colored blue (cells), grey (RbmA), red (RbmC), and green (Bap1). (B) 3D biofilm architecture with colours as in (A). Adjusted from Berk et al. (2012) (Berk et al. 2012). Permission for using the image was granted.

The spatial visualization of molecular species in bacterial biofilm CLSM focuses mainly on protein analysis. These studies use antibody labels to follow a specific protein in live bacteria. Localizing proteins in biofilms can provide information on biofilm formation and biofilm-surface interactions. For example, Berk et al. used CLSM to visualize the essential matrix proteins, RbmC and Bap1, produced during the biofilm formation along with polysaccharides (Berk et al. 2012). Labelling these proteins allowed the 2D and 3D investigation of the molecular mechanisms involved in the biofilm formation of *Vibrio cholerea* biofilms (Figure 7). However, the spatial resolution of CLMS was insufficient to study intermediate steps in the 3D biofilm development. Therefore, Berk et al. (2012) constructed a multi-colour 3D super-resolution imaging apparatus using stochastic optical reconstruction microscopy (STORM). This technique relies on individual activation of fluorophores labelled molecules by stochastic activation. The fluorophores will switch between an off state and an activated state, thereby emitting light. The STORM measurements produced a localization precision of 19, 21, and 42 nm in X, Y, and Z, respectively. Furthermore, molecular images of the biofilm formation and adhesion-related protein LapA were obtained using fluorescence imaging by El-Kirat-Chatel et al. (2014). The images showed the distribution of LapA at the cell surface and the protein accumulation in mutated cells. Lastly, Decker et al. investigated the spatial distribution of a novel 18 kDa small basic protein (Sbp) using CLSM (Decker et al. 2015). Sbp was predominantly identified in the biofilm matrix in a heterogeneous manner. The protein accumulated in unevenly spread clusters and was mainly concentrated within the biofilm-surface interface.

CLSM has been the standard, well-established technique for three-dimensional *in situ* biofilm visualization for decades. However, the specificity of CLSM is limited to the specificity of the fluorescent probe labels that can be used, as non-specific binding induces a background signal. The number of simultaneously detectable probes limits the parallel imaging of analytes (Lanni et al. 2014). Furthermore, labelling techniques are not preferred when imaging the spatial distribution of small molecules. The addition of the antibody probe may influence the mechanism of the molecules and, therefore, their spatial distribution (Ding et al. 2016). However, new developments in nanobody labelling increase the precision of the molecular spatial distribution by using smaller labelling tags, showing the potential of this technique in biofilm research (Melia et al. 2021).

### Micro-CT

µCT is a commonly known technique in clinical practice and uses X-rays to recreate a 3D image of the object on a micro-scale. Keren-Paz et al. showed that high-resolution µCT provides structural insight into the calcium structures present within the biofilm and allows investigation of the calcium-carbonated areas within biofilms and their effect on the diffusion of small molecules (Keren-Paz et al. 2018). Furthermore, they found that a mechanism for the high antibiotic resistance of biofilms involves the formation of extracellular calcium carbonate sheets that serve as diffusion barriers protecting the colonies. The 3D distribution of calcium carbonate in biofilms can reduce the diffusion of small molecules throughout the biofilm by several orders of magnitude when compared to gels or fiber-like materials. By using X-ray technologies to image biofilms in medically relevant settings, it may be possible to predict antibiotic diffusion within biofilms.

### ^19^F nuclear magnetic resonance oximetry

One paper in this review presented ^19^F nuclear magnetic resonance (NMR) oximetry as a technique for obtaining molecular information in a biofilm. ^19^F NMR oximetry uses exogenously administered reporter molecules to quantitatively measure oxygen tension in a tissue or fluid, an interesting outcome parameter to gain insight into biofilm function and metabolism. Oxygen availability is one of the most critical parameters governing microbial and biofilm growth behaviour but is complex or, in some cases, intractable to measure. The biofilm is a metabolic heterogeneous structure, and metabolically distinct subzones can be divided according to oxygen availability (Liu et al. 2019). To track metabolic activity, relevant when researching anti-biofilm drug delivery, for example, spatially mapping oxygen availability is a valuable outcome parameter. Furthermore, to gain insight into biofilm activity, the relation between the arrangement of structural components and mass transfer must be understood (de Beer et al. 1994). Simkins et al. showed the effectiveness of ^19^F NMR oximetry in measuring oxygen distribution in microbial and biofilm systems without affecting oxygen transport (Simkins et al. 2018). The technique spatiotemporally tracks oxygen concentration in dynamic, complex systems and can extract essential parameters, such as diffusion coefficient. Furthermore, combining fluid flow and oxygen transport information allows for the generation of a spatial map of bacterial growth rate.

### Multimodal imaging

**Figure 8.**
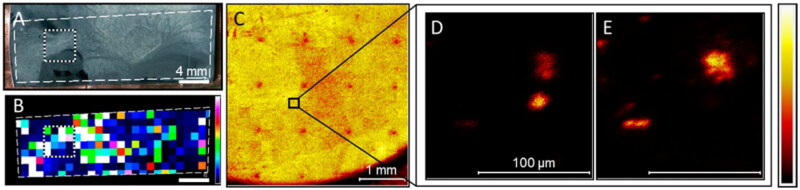
MALDI-guided SIMS results of *Pseudomonas aeruginosa* biofilm. (A) Optical (B) MALDI ion images of PQS (*m/z* 260.17). (C) SIMS total ion image showing laser ablation marks in the sample. These points are correlated with the MALDI ion image to select a region of interest. (D,E) SIMS imaging at the ROI visualizes the localization of (D) PQS (*m/z* 260.17) and (E) HHQ (*m/z* 244.17) (Lanni et al. 2014). Permission for using the image was granted.

Multimodal imaging combines two or more imaging modalities to gather information on the same specimen (Walter et al. 2020). By providing complementary information about the sample, such as molecular information, structure, function, and dynamics, more in-depth knowledge of biofilm characteristics or processes within the biofilm can be gained. Lanni et al. and Baig et al. presented a Confocal Raman microscopy (CRM)/SIMS correlated workflow and demonstrated how their complementarity information could be exploited for enhanced molecular imaging of a biofilm (Lanni et al. 2014; Baig et al. 2015). The correlation of MSI and CRM data enabled the broad characterization of the chemical composition of the biofilm microenvironment as well as specific constituent analytes, including quinolones, which are a class of signalling molecules involved in *P. aeruginosa* biofilm growth and maturation. However, this multimodal approach is not straightforward, as the precise correlation of the images acquired by two different instruments is complicated. To overcome this problem, Lanni et al. developed a chemical microspot-based system for navigation purposes to align the imaging data (Lanni et al. 2014). The nanometer-scale spatial resolution provided by CRM is complemented by the chemical specificity of the correlated SIMS data. By combining these two modalities, nine quinolones, and additional related metabolites were detected. Baig et al. (2016) presented another multimodal approach combining CRM and SIMS, where CRM, combined with principal component analysis, was first used to identify broad molecular classes. This information was used to guide the MSI analysis (Baig et al. 2015). With this approach, isomeric analytes can be distinguished, which is impossible with CRM or MS alone. Baig et al. (2016) distinguished two isomeric QS molecules in a quinolone-rich region of a biofilm. QS molecules are crucial for early biofilm formation and the growth and organization of biofilms. As stated, altering or blocking QS pathways or molecules can potentially lead to new biofilm treatment strategies, underscoring the need for multimodal approaches to image complex biological systems. MALDI was used in multimodal approaches in combination with SIMS and fluorescence imaging (Lanni et al. 2014; Si et al. 2016). The combination of MALDI with fluorescence imaging enabled the comparison of the spatial distribution of selected molecules in association with protein expression. This approach revealed information on cellular heterogeneity and function, which were not obtained using single imaging methods. Si et al. used MALDI and fluorescence imaging to compare metabolite distribution to spatial patterns of differentiated cells, using MALDI MSI for chemical mapping and fluorescence imaging for protein visualization (Si et al. 2016). These molecules were detected in distinct populations of biofilm cells, which were previously assumed as identical regions. Dunham et al. sequentially imaged a biofilm using SIMS, followed by MetA-SIMS and MetA-LDI (Dunham et al. 2016). The small molecule imaging capabilities of the three techniques were compared, and it was shown that metallization is a recommended sample treatment for small molecule imaging of biofilms, as it showed a dramatic reduction in background noise. MetA-SIMS is recommended when intact molecular ions must be analyzed with high spatial resolution. Lanni et al. combined MALDI and SIMS in a MALDI-guided SIMS approach for imaging *P. aeruginosa* (Figure 8) (Lanni et al. 2014). In this approach, MALDI was used to obtain a low-resolution molecular map, after which SIMS was used for high-resolution imaging of metabolites.

## Research application purposes and recommendations

### Biofilm formation and maturation

The different phases of biofilm formation can be studied in a spatiotemporal manner by in-depth analysis of biomolecules related to adhesion and growth, such as lipids and proteins. SIMS and MALDI are the main techniques for this application. SIMS, however, is limited to the detection of small molecules but has a higher resolution, whereas with MALDI, larger molecules can be imaged but a lower lateral resolution is reached as it suffers from ion interference of the MALDI matrix molecules, laser spot size, and matrix crystal size. Multiple laser desorption ionization techniques can be employed to eliminate the use of MALDI matrices, including IR-MALDI, Fs-LDPI, and NIMS. These methods, however, require further optimization. They often lack sensitivity and are highly selective. Post-ionization strategies like MALDI-2 offer enhanced sensitivity and low chemical background. The analysis of different molecular classes can be improved using MALDI-2, making it a promising technique for future biofilm research. To image the elemental composition of biofilm and biofilm formation at subcellular resolution, SIMS should be the technique of choice. However, the required dehydration steps can alter the sample structure. Cryo-Orbi-SIMS is a promising SIMS mode that enables the imaging of hydrated biofilms. In cryo-SIMS, the sample is imaged in a frozen state, preserving the morphology. Besides MSI, CLSM can be used to study micro-processes of biofilm formation and maturation but is limited to the specificity of the labelling probe. Furthermore, due to the fluorescence principle, biofilm matrix molecules cannot be imaged simultaneously. In addition, SERS is a valuable technique to image molecules during biofilm formation, even in low concentrations. The technique is non-invasive, non-destructive, and allows *in situ* imaging with low water interference. However, reference spectra of known molecules must be available.

**Table 3.** Imaging methods with the recommended research applications.

Method	Research application				
	Biofilm growth (formation and maturation)	Cell-material interaction	Cell-cell signalling	Environmental stress and drug delivery	Cell metabolism
**MSI**					
SIMS	X	X		X	X
MALDI	X	X			
NIMS	X	X			
**NanoDESI**		X			
**Fluorescence imaging**					
CLSM	X	X	X	X	
**Spectroscopy**					
SERS	X		X	X	
SR-FTIR			X		
**µCT**					X
**NMR**					X

### Cell material interaction

When investigating biofilm formation or maturation on a specific substrate material, it is essential to consider that SIMS allows for higher freedom of substrate material, whereas MALDI is often restricted by conductive sample substrates. This limits the imaging of cell-material interaction with MALDI and therefore limits the analysis of larger biomolecules. This limitation, however, can be overcome by culturing the biofilm onto modified conductive surfaces to research the cell-material interaction. Furthermore, DESI can be used to visualize cell-material interactions since DESI is not limited to conductive sample substrates. However, the spatial resolution of DESI needs to be improved. Finally, as SIMS is limited to small molecules, fluorescence can be helpful to image larger molecules on various substrate materials, although the possibility of auto-fluorescence by the material must be kept in mind. Multiple other confocal microscopy techniques that were not found in the included literature, could be employed to study cell-material interactions. These techniques will be discussed below.

### Cell-cell signalling

When studying cell-cell signalling, the molecules of the highest interest are QS molecules, such as PYO and PQS. Spectroscopic techniques, including SR-FTIR and SERS, are used to spatially identify QS molecules in bacterial biofilms. SR-FTIR enables high-resolution, label-free analysis of *in situ* biofilms but suffers from high water interference. Therefore, Raman spectroscopy, specifically SERS, is the primary technique to map QS molecules. As stated, SERS is non-invasive, non-destructive, and highly specific with low water interference. Also, this review showed that cell-cell signalling could be imaged in multi-species biofilms using MALDI by visualizing rhamnolipids and quinolones.

### Environmental stress and drug delivery

To optimize or develop new biofilm treatment strategies, it is essential to image the biofilm under environmental stress, in the presence of drugs, and on different surfaces, including antimicrobial material technologies. All the discussed techniques can visualize the general presence of molecules and biofilm structure and thus see how environmental stressors affect the biofilm’s chemical and morphological characteristics. Specifically, molecular imaging techniques are valuable for tracking drug delivery in a biofilm or visualizing the penetration depth of substances in the biofilm to better understand metabolic processes in the biofilm. For this application, SIMS, CLSM, and SERS are valuable techniques. However, with the latter, the resonance wavelength of the drug must be known. With SIMS, endogenous and exogenous biofilm compounds can be simultaneously imaged, giving valuable biochemical information on stress response and drug delivery. Furthermore, with both SIMS and CLSM, the penetration of molecules, such as drugs, can be tracked by 3D imaging. However, it must be kept in mind that the fluorescent labels used with CLSM, can interfere with drug release processes. NanoDESI and SIMS are valuable techniques for imaging metabolic processes and metabolic profiling without labelling. Visualizing metabolic processes and molecules can offer a deeper understanding of drug uptake and effect, as biofilms are metabolically heterogeneous.

### Biofilm imaging techniques—not included in the systematic search

Multiple non-molecular imaging techniques that were not included in this review can be used to image a biofilm. These techniques do not visualize the biofilm on the level of a molecular group nor give molecular information but are primarily used to visualize the presence and morphology of a biofilm. Also, some techniques were not included in the literature search due to the lack of application in clinical biofilm research. Some popular methods include AFM, SEM, NMR, X-ray, MRI, PET-CT, and NIR. Furthermore, laser ablation inductively coupled plasma MS (LA-ICP) might be an interesting method for molecular imaging of biofilms and yields future potential. LA-ICP can spatially quantify trace elemental distribution and isotope within biological tissue sections (Latimer et al. 2009). Therefore, by following essential elements, LA-ICP can be employed to analyze bacterial metabolism. LA-IPC was not included in the literature search and, thus, not described within this systematic research. Other techniques of interest for the biofilm research field that were not included in the systematic search are discussed below.

#### Alternative optical imaging methods

##### Light sheet microscopy/single-plane illumination microscopy

Besides CLSM, new advanced microscopic techniques are developed to obtain molecular information. An interesting example is the combination of fluorescence correlation spectroscopy (FCS) with single-plane illumination microscopy (SPIM). This method enables the 3D detection of diffusion maps in whole cross sections with limited photo damage of the sample. Sankaran et al. used FCS-SPIM to determine the molecular diffusion coefficient in *P. aeruginosa* biofilms (Sankaran et al. 2019). Measurement of molecular diffusion is of interest since it is commonly linked to multiple biofilm characteristics, such as nutrient trapping, antibiotic tolerance, and signal accumulation. Therefore, measuring the diffusion coefficient can provide information on the influence of the biofilm microenvironment on the mobility of molecules.

##### Single-molecule localization microscopy

Single-molecule localization microscopy (SMLM) is one of the main categories of super-resolution microscopy (Lelek et al. 2021). The SMLM techniques are based on the fact that a single fluorescence molecule can be spatially detected if the point spread function does not overlap. To avoid this overlap, the emission of the individual fluorescence molecules is separated in time. The most common approach is photoswitching, in which the fluorescence molecules can be switched “on” and “off.” Various approaches can regulate this event, such as laser irradiation or adjusting the chemical environment. One of these techniques is fluorescence photo-activated localization microscopy (PALM), where UV light can activate fluorescence proteins. Another interesting approach is STORM, which was mentioned previously, as Berk et al. employed this technique to visualize the distribution of RmbC in a biofilm (Berk et al. 2012). STORM uses synthetic fluorophores that can be regulated by changing the chemical environment with suitable buffers. Finally, Point accumulation in nanoscale topography (PAINT) does not use the photo-switching principle but relies on binding the dye with the target (Jimenez et al. 2020).

##### Mesolens

Optical microscopy has been used to investigate channel features in the biofilm. For example, a study showed that a *Pseudomonas* biofilm folds to increase oxygen transport when the biofilm reaches a certain mass (Kempes et al. 2014). However, studies applying optical microscopy to visualize the biofilm have shown that either bacteria can be imaged individually with a high-power objective lens or the biofilm structure can be visualized at low magnification with poor depth resolution, limiting visualization of individual bacteria (Rooney et al. 2020). To overcome this, Mesolens can be used to image biofilms *in situ* with sub-cellular resolution. The Mesolens is a large objective with 4× magnification with a numerical aperture of 0.47, combining the low magnification with a high numerical aperture resulting in a lateral resolution of 700 nm and an axial resolution of 7 µm. Mesolens was used to investigate *Escherichia coli* biofilms and undocumented channel systems were found, thereby gaining insight into biofilm organization, nutrient distribution systems, and ECM component distribution.

##### Multiphoton microscopy

Multiphoton microscopy is a powerful tool for imaging cellular and subcellular events *in situ*. Multiphoton microscopy uses long excitation wavelengths, which is responsible for deeper penetration than single-photon microscopy, as light scattering declines rapidly with an increasing wavelength, especially in a dense scattering matrix as a bacterial biofilm (Cho et al. 2011). The technique has been used to visualize the unique social motility of *Flavobacterium johnsoniae*, where a rotary gliding motor in the cells moves motility adhesins around the cell to generate movement (Li et al. 2021).

##### Spinning disc microscopy

Spinning disc microscopy has been used to gain insight into the adhesion processes of *Xylella fastidiosa*, a bacterial plant pathogen (Janissen et al. 2015). The adhesion of bacteria is the first crucial step in biofilm formation. Using spinning disc microscopy, temporal resolution was improved compared to CLSM.

##### Alternative confocal microscopy techniques

With CLSM, alternative methods are present that could be valuable in biofilm research regarding cell-cell signalling, for example, the use of responsive indicator dyes measuring electrical signalling. A study used a fluorescent cationic dye thioflavin to quantify membrane potential within a biofilm, and a function for ion channels in bacterial biofilms has been demonstrated (Prindle et al. 2015). Furthermore, other techniques are present that could be valuable in biofilm research regarding cell-cell signalling. Bioluminescence resonance energy transfer (BRET) involves resonance energy transfer between a bioluminescent donor and a fluorescent acceptor. The donor emits photons intrinsically, and therefore fluorescent excitation is unnecessary. This way, BRET overcomes problems regarding photobleaching and autofluorescence that are encountered with fluorescence resonance energy transfer (FRET) (Xu et al. 2007).

##### Hyperspectral imaging

Hyperspectral imaging allows two-dimensional imaging by acquiring across a wide range of the electromagnetic spectrum. The spectral information of the imaged object reflects its identity and composition, combined with spatial information. The technique is applied in various research applications and industries, including biofilm research. Hyperspectral imaging can be used to research biofilm growth dynamics in a non-invasive manner. Previously, hyperspectral imaging stimulated Raman scattering microscopy to visualize the interplay between the antibiotic vancomycin and *S. aureus* biofilm to gain insights into the resistance mechanism (Bae et al. 2019).

##### Scanning transmission X-ray microscopy

Scanning transmission X-ray microscopy (STXM) can provide spatial information on macromolecular distribution in bacterial biofilm cells, including the distribution of proteins, lipids, saccharides, carbonates, and nucleic acids. STXM is a powerful technique that uses near-edge X-ray absorption spectroscopy (NEXAFS) and can be applied to fully hydrated samples due to the ability of X-ray to penetrate water, making it highly suitable in biofilm analysis (Benzerara et al. 2004). In STXM analysis, an X-ray beam is focussed on a spot, scans the sample, and the transmitted X-ray intensities are analyzed per sample location, providing spatial information down to 50 nm. X-ray microscopy offers information on nearly all elements and provides chemical composition mapping based on the bonding structure.

##### Correlative light-electron microscopy

Correlative light-electron microscopy (CLEM) provides complementary information on a sample by combining electron microscopy (EM) and light microscopy. Light microscopy provides spatial information on, for example, live cell dynamics using fluorescence labels (Vicidomini et al. 2010). However, the spatial resolution is limited by light diffraction down to 50 nm resolution with super-resolution techniques. Therefore, further improvements in resolution can be achieved by applying EM. EM can provide images at the molecular level and reveals non-labelled structures, such as membranes, macromolecules, and organelles. CLEM has been used to visualize biofilm-associated *P. aeruginosa* markers involved in antibiotic resistance mechanisms (Kumar et al. 2022).

##### Topography and recognition imaging

Topography and recognition imaging (TREC) is an imaging technique based on AFM that records recognition and topography images. In TREC, an AFM tip is functionalized with a chemical group or ligand and scans the sample (Zhang et al. 2019). Then, a specific amplitude is applied to the tip, which changes correlate to the particular binding event between the functionalized tip and the substrate. Since the functionalization on the tip only interacts with specific molecules, the created images visualize individual target molecules and are called recognition images.

## Conclusion

A fundamental understanding is needed to reveal the underlying mechanism of biofilm formation, behaviour, and response to anti-biofilm treatments. Imaging techniques can visualize the chemical distribution of metabolites, lipids, peptides, and proteins in bacterial biofilms. We provide an overview of the literature that gained insight into biofilm composition, molecular interactions, and structural knowledge of the biofilm. Here, we performed a systematic literature review to get an overview of imaging techniques used to image bacterial biofilm molecularly. We evaluated current molecular imaging techniques used in literature to generate these molecular images, including mass spectrometry-based, fluorescence labelling, spectroscopic, NMR, µCT, and multimodal approaches. The general working mechanism of each technique is explained, and the advantages and disadvantages of molecular imaging of biofilms are highlighted. Recommendations are offered in this review for each research application and desired molecular groups. However, the preferred imaging technique depends on the research question to be answered and the research methodology regarding the type of biofilm, substrate material, and desired resolution. Emphasis must be placed on the significant potential of multimodal imaging; combining the advantages of each technique leads to great insight into the chemical composition and processes of the biofilm and possibilities for enhanced biofilm prevention or treatment strategies. Future research is necessary to decrease the complexity of the methods and data processing. Each imaging technique has specific strengths in different research applications, which were elaborated on. It was recommended what imaging technique to use when imaging a biofilm for a research question related to biofilm formation, cell-cell communication, cell-material interaction, or the effect of environmental stress and drugs. A significant step forward in biofilm research for preventing or eradicating biofilm-related infections can be found in molecular imaging techniques of the biofilm.

## Supplementary Material

Supplemental Material

## Data Availability

All data is provided in the current document. **Box 1.** Advantages and disadvantages of each molecular imaging modality.

**Table:** 

**SIMS**	+ High spatial resolution, 3D imaging possible, high freedom of sample substrate
	− High fragmentation, limited molecular coverage
Solid state	− Dehydration step affects native biofilm structure
Liquid-state	+ Biofilm analysis in native state
	− Low signal intensity
	
**MALDI**	+ Minimal fragmentation, wide molecular coverage
	− Matrix application: reduces sensitivity and increases background signal
	− Spot-to-spot variability due to noise, saturation, sample charging
	− Restricted freedom of sample substrate
**Enhanced LDI methods**	
MALDI-2	+ Enhanced spatial resolution and ionization efficiency
IR-MALDI-2	+ Reduction in background, broad range of analytes
Fs-LDPI	+ Elimination matrix application, Minimal sample damage for same-spot analysis
MetA-LDI	+ Elimination of matrix application, Enhanced ionisation efficiency
NIMS	+ Elimination matrix application
	
**NanoDESI**	+ Minimal sample preparation required
	− Availability
	
**SERS**	+ Non-destructive and non-invasive, High specificity, Low water background
	− Limited sensitivity, targeted analysis for molecule identification needed
	− Surface enhancement needed as biofilms have weak Raman scattering
	
**SR-FTIR**	+ High resolution, label free
	− High water background
	
**Fluorescence**	+ High sensitivity, 3D imaging possible, quantitative analysis possible
	
**Microscopy**	− Limited to specificity fluorescent probe labels
	− Background noise due to non-specific label binding
	
**NMR**	+ Unique spatial mapping of oxygen
	− Limited application in molecular biofilm research
	
**µCT**	+ Unique 3D distribution of calcium in biofilm
	− Limited application in molecular biofilm research

## Registration and protocol

Open science framework

https://doi.org/10.17605/OSF.IO/ERJKX
