# Supplementary material for: Molecular imaging of bacterial biofilms—a systematic review
Source: Crit Rev Microbiol. 2023 Jul 15;50(6):971–92. doi: 10.1080/1040841X.2023.2223704 (PMC11523921; doi:10.1080/1040841X.2023.2223704)
Supplement: Supplemental Material [file IMBY_A_2223704_SM3899.docx]

**Table S1** – EMBASE search string

| Search Number | Search term |
| --- | --- |
| 1 | molecular imaging/ |
| 2 | biofilm/ |
| 3 | biofilms/ |
| 4 | 2 OR 3 |
| 5 | 1 AND 4 |
